# Turnour necrosis factor stimulates endothelin-1 gene expression in cultured bovine endothelial cells

**DOI:** 10.1155/S0962935192000401

**Published:** 1992

**Authors:** Silvia Orisio, Marina Morigi, Carla Zoja, Norberto Perico, Giuseppe Remuzzi

**Affiliations:** 1 Mario Negri Institute for Pharmacological Research Via Gavazzeni 11 Ospedali Riuniti di Bergamo Bergamo 24100 Italy; 2 Division of Nephrology Ospedali Riuniti di Bergamo Bergamo 24100 Italy

## Abstract

We have studied the effect of human recombinant tumour necrosis factor-α (TNF-α) on gene expression and production of endothelin-1 in cultured bovine aortic endothelial cells. TNF-α (10 and 100 ng ml^−1^) increased in a time dependent manner the preproendothelin-1 mRNA levels in respect to unstimulated endothelial cells. TNF-α induced endothelin-1 gene expression was associated with a parallel increase in the release of the corresponding peptide in the culture medium. These findings suggest that the enhanced synthesis and release of endothelin-1 occurring in conditions of increased generation of TNF, may act as a modulatory factor that counteracts the hypotensive effect and the excessive platelet aggregation and adhesion induced by TNF.


Research Paper

Mediators of Inflammation, 1, 263–266 (1992)

### Tumour necrosis factor stimulates endothelin-1 gene expression in cultured bovine endothelial cells

Silvia Orisio,[1] Marina Morigi,[1] Carla Zoja[1]

Norberto Perico[1,2] and

Giuseppe Remuzzi[1,2CA]

[1]Mario Negri Institute for Pharmacological Research, Via Gavazzeni 11 and [2]Division of Nephrology, Ospedali Riuniti di Bergamo, 24100 Bergamo, Italy

[CA] Corresponding Author

**We have studied the effect of human recombinant tumour necrosis factor-α (TNF-α) on gene expression and production of endothelin-1 in cultured bovine aortic endothelial cells. TNF-α (10 and 100 ng $ml^{-1}$) increased in a time dependent manner the preproendothelin-1 mRNA levels in respect to unstimulated endothelial cells. TNF-α induced endothelin-1 gene expression was associated with a parallel increase in the release of the corresponding peptide in the culture medium. These findings suggest that the enhanced synthesis and release of endothelin-1 occurring in conditions of increased generation of TNF, may act as a modulatory factor that counteracts the hypotensive effect and the excessive platelet aggregation and adhesion induced by TNF.**

**Key words:** Endothelial cells, Endothelin, Gene expression, Tumour necrosis factor

### Introduction

Humoral, neural and mechanical stimuli can induce endothelium dependent vasoconstriction through the release of vasoactive substances from endothelial cells.[1]

Recently, the endothelins, a family of 21-aminoacid peptides isolated and purified from endothelial cells,[2] have been recognized as potent vasoconstrictors of mammalian blood vessels.[3] Stimulation of preproendothelin-1 gene expression in cultured endothelial cells by thrombin, transforming growth factor β, interleukin-1 and shear stress with subsequent release of the active peptide in the culture medium,[2,4–6] implies that endothelial cell perturbation or injury may be associated with a local release of endothelin that is likely to play an active role in the process of tissue injury and repair. This is in keeping with the recent discovery that endotoxin, a noxious agent for endothelial cells,[7,8] stimulates endothelin release *in vitro* and *in vivo*.[9]

Studies in the last few years have convincingly documented that most of the effects of endotoxin infusion into experimental animals are mediated by tumour necrosis factor (TNF), a macrophage-derived peptide with potent biological activities.[10,11] When infused into normal rabbits TNF induced glomerular changes remarkably similar to those found in animals given endotoxin,[12] that included endothelial cell damage, polymorph accumulation and fibrin formation.[13]

The present study was designed to investigate whether human recombinant TNF-α modifies preproendothelin-1 gene expression in cultured bovine aortic endothelial cells, and whether changes in endothelin gene expression are associated with the release of the corresponding peptide in the cell supernatant.

### Materials and Methods

*Endothelial cell culture and incubation:* Bovine aortic endothelial cells were obtained by collagenase digestion and cultured as previously described.[14] Cells were identified as endothelial by their cobblestone appearance in monolayer and the presence of factor VIII antigen by using indirect immunofluorescence microscopy.[15]

Confluent cells (passage 1–2) grown in 100 mm plastic dishes were incubated for 1, 2 or 6 h at 37°C in 5 ml D-MEM (Gibco, Grand Island, NY) with 1% foetal calf serum (Hyclone, Logan, UT) in the absence or presence of human recombinant TNF-α (10 and 100 ng $ml^{-1}$). Human recombinant TNF-α (Cetus Corp., Emeryville, CA) had a specific activity of $10^7$ U $mg^{-1}$ of protein and was virtually endotoxin free (<0.3 pg endotoxin $\mu g^{-1}$ by the Limulus amoebocyte lysate assay). At the end of the incubation, the supernatants were collected for endothelin-1 measurement and the cells were used for total cellular RNA preparation.

*Preparation of total cellular RNA and Northern blot analysis:* Total cellular RNA was isolated from endothelial cells by lysing cells in guanidium isothiocyanate and recovering RNA by centrifugation through caesium chloride.[16] RNA (10 μg) was then fractionated on a 1.2% agarose gel with 6% formaldehyde and blotted onto synthetic membranes (Gene Screen

© 1992 Rapid Communications of Oxford Ltd

Plus, New England Nuclear).[17] Human preproendothelin-1 probe,[17] was labelled to a specific activity of $10^9$ cpm $\mu g^{-1}$ by using hexanucleotide primers and $^{32}$P-dCTP.[18] Hybridization was performed for 20 h at 60°C[17] and the membranes were washed as previously described.[17] Membranes were subsequently rehybridized with a mouse α-actin cDNA[19] to determine an internal standard of total RNA content. Following optimal exposure, the autoradiographs of each experiment were scanned by a laser densitometer in order to quantify the relative amounts of radioactively labelled probe bound for each transcript. Endothelin mRNA optical density was normalized to that of the constituently released mouse actin gene expression.

*Radioimmunoassay for endothelin:* Unextracted samples were assayed at appropriate dilutions in phosphate buffer (pH 7.2) containing 0.1% Triton X-100 and 0.3% albumin (RIA buffer) as previously described.[17] Results were expressed as pg $10^{-6}$ cells. The minimum detectable concentration that could be measured was 0.4 pg per tube. The cross reactivity of the antibody with other endothelins is as follows: endothelin-2, 46.9%; endothelin-3, 17%; and big endothelin-1, 9.4%.[17]

*Statistical analysis:* Results of endothelin production are expressed as mean ± SD. Statistical analysis was performed by using two-way analysis of variance by Tukey's test. Statistical significance level was defined as $p < 0.05$.

### Results and Discussion

We first determined whether the expression of the preproendothelin-1 gene in bovine aortic endothelial cells is stimulated by TNF-α. To this purpose we monitored by Northern blot analysis endothelin-1 mRNA levels in endothelial cells exposed for 1, 2 and 6 h to two different concentrations of TNF-α. As shown in Figure 1 (panel A), TNF-α at the concentration of 10 ng $ml^{-1}$ increased in a time dependent manner the preproendothelin-1 mRNA levels in respect to unstimulated endothelial cells. Analysis of the optical density of the autoradiographic signals (Figure 1, panel B) indicated an increase of preproendothelin-1 mRNA levels ranging 1.9–2.6 fold over the basal levels within 6 h. Incubation of endothelial cells with a higher concentration of TNF-α, 100 ng $ml^{-1}$, reproduced the pattern observed with 10 ng $ml^{-1}$ TNF-α without a further stimulation of endothelin-1 transcript (Figure 2, Table 1).

Next we sought to establish whether the increase of endothelin gene expression elicited by TNF-α in bovine aortic endothelial cells was associated with

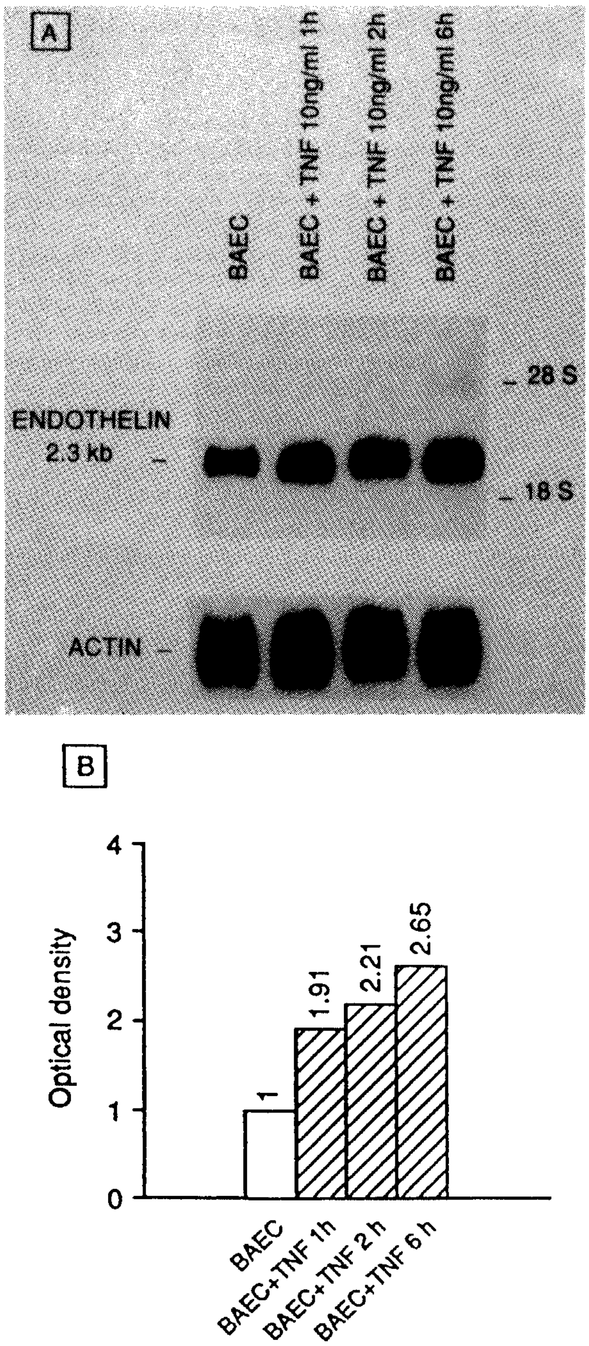

FIG. 1. Panel A: Endothelin-1 mRNA expression in BAEC incubated for 1, 2 and 6 h with medium alone or 10 ng $ml^{-1}$ TNF-α. The same membrane was rehybridized to the actin probe. Blots are representative of four individual experiments. Panel B: Corresponding densitometry of the autoradiograph reported in Panel A. The optical density of the autoradiography signals was quantitated and calculated as the ratio of endothelin to actin mRNA. The mRNA level of unstimulated BAEC (control) was assigned the number 1.0 and all other values were calculated relative to that value.

increased synthesis and release of the endothelin-1 peptide in the cell supernatants. Figure 3 shows endothelin-1 production, measured by a specific radioimmunoassay, in supernatants of endothelial cells incubated for 1, 2 and 6 h with medium alone (control) or TNF-α at concentrations of 10 and 100 ng $ml^{-1}$. Endothelin-1 production in supernatants of endothelial cells exposed to TNF-α for

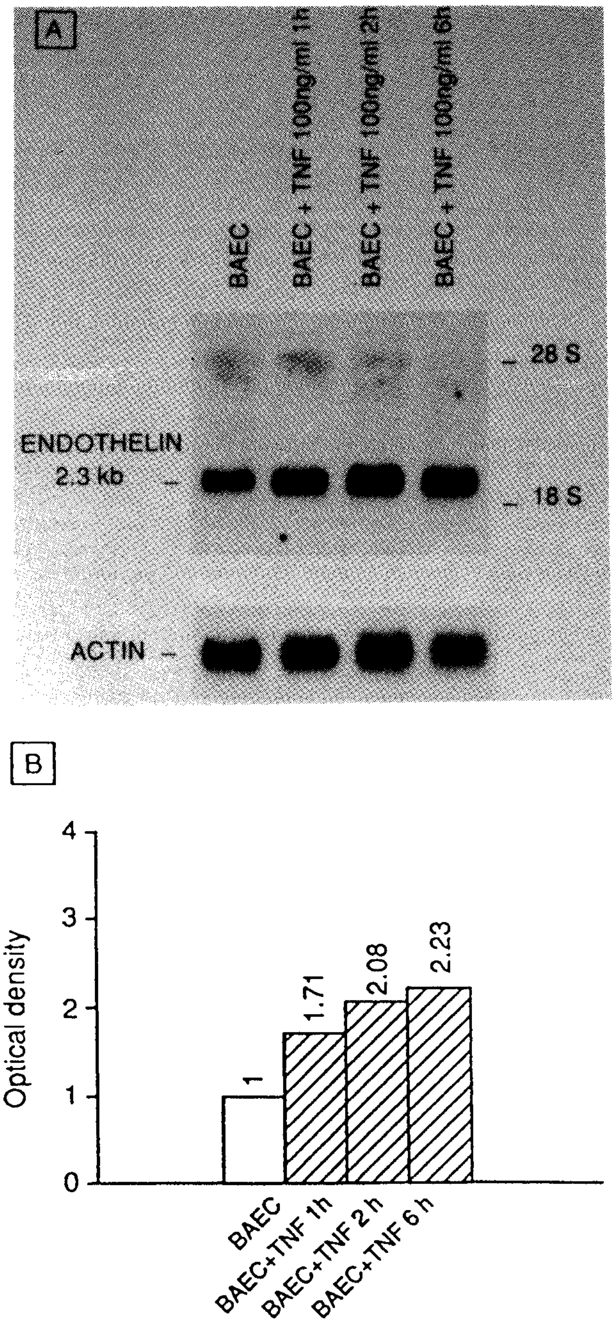

FIG. 2. Panel A: Endothelin-1 mRNA expression in BAEC exposed for 1, 2 and 6 h to medium alone or 100 ng $ml^{-1}$ TNF-α. Blots are representative of four individual experiments. Panel B: Corresponding densitometry of the autoradiograph reported in Panel A.

**Table 1.** Endothelin gene expression in bovine aortic endothelial cells after TNF stimulation

| | Incubation time | | |
|---|---|---|---|
| | 1 h | 2 h | 6 h |
| TNF 10 ng/ml | 1.92 ± 0.14 | 2.18 ± 0.31 | 2.59 ± 0.34 |
| TNF 100 ng/ml | 1.72 ± 0.10 | 2.02 ± 0.14 | 2.55 ± 0.30 |

Values are means ± SD ($n = 4$).
The ratio of endothelin/actin optical density of four individual experiments was calculated relative to endothelin/actin ratio in unstimulated cells. Control ratio was assigned the number 1.0 and all other values were calculated relative to that value.

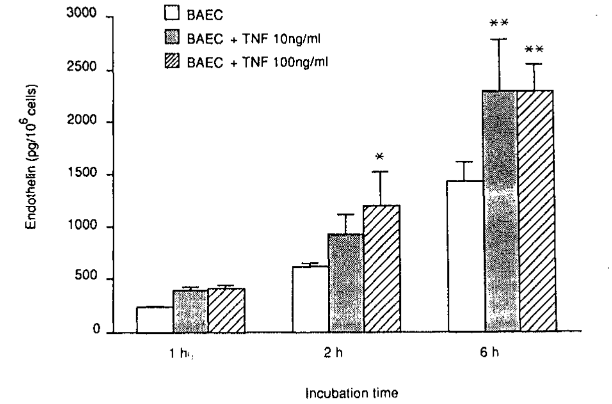

FIG. 3. Endothelin-1 production in supernatants of BAEC incubated for 1, 2 and 6 h with medium alone (control) or TNF-α (10 and 100 ng $ml^{-1}$). Endothelin measured in a media blank was 0.5 ± 0.2 pg $ml^{-1}$. Values are expressed as mean ± SD.*$p < 0.05$; **$p < 0.01$ versus control BAEC.

1 h showed a trend to increase compared to unstimulated cells. After 2 h incubation of endothelial cells with 10 ng $ml^{-1}$ TNF-α, endothelin-1 production was numerically higher than control values, but the difference did not reach statistical significance. The level of endothelin-1 increased to a statistically significant ($p < 0.05$) extent when endothelial cells were exposed for 2 h to 100 ng $ml^{-1}$ TNF-α, as compared with cells incubated with medium alone. A further and significant ($p < 0.01$) increase in endothelin production in respect to unstimulated cells was observed when endothelial cells were incubated with TNF-α for 6 h, without significant differences between 10 or 100 ng $ml^{-1}$ TNF-α.

Altogether these findings show that TNF-α increases endothelin-1 gene expression in bovine endothelial cells and that TNF-α induced endothelin-1 gene expression is associated with a parallel increase in the release of the corresponding peptide in the culture medium. The apparent weak action of TNF-α, as compared to other stimuli, suggests that this cytokine acts as a mild transcriptional activator of the endothelin-1 gene.[4–6]

The central role of TNF-α in the pathogenesis of septic shock and the capacity for TNF-α to induce profound alterations of endothelial cell function has been well documented.[20] It has been recognized recently that the endothelium contributes to the control of vascular tone by releasing vasodilators, including endothelium-derived relaxing factor (EDRF), nitric oxide (NO),[21] and vasoconstrictors such as endothelin. Thus, TNF-α induces NO synthase activity in bovine aortic endothelial cells in culture, and the consequent release of NO,[22] which largely accounts for hypotension[23–25] and acute renal failure[13] induced by TNF *in vivo*. The release of endothelin observed after exposure of endothelial cells to TNF-α may be considered an additional modulator of vascular tone at a systemic[26,27] and renal level.[28–30]

On the other hand endothelin, besides its potent vasoactive properties also inhibits local platelet aggregation *in vivo*.[30] It is therefore conceivable that the enhanced synthesis and release of endothelin in response to TNF contribute to counteracting excessive platelet activation in the microcirculation upon challenge with stimuli, such as endotoxin, that promote the release of TNF.

### References

1. Brenner BM, Troy JL, Ballerman BJ. Endothelium-dependent vascular responses. *J Clin Invest* 1989; **84:** 1373–1378.
2. Yanagisawa M, Kurihara H, Kimura S, *et al.* A novel potent vasoconstrictor peptide produced by vascular endothelial cells. *Nature* 1988; **332:** 411–415.
3. Inoue A, Yanagisawa M, Kimura S, *et al.* The human endothelin family: three structurally and pharmacologically distinct isopeptides predicted by three separate genes. *Proc Natl Acad Sci USA 1989;* **86:** 2863–2867.
4. Kurihara H, Yoshizumi M, Sugiyama T, *et al.* Transforming growth factor-$\beta$ stimulates the expression of endothelin m-RNA by vascular endothelial cells. *Biochem Biophys Res Commun* 1989; **159:** 1435–1440.
5. Yoshizumi, M, Kurihara H, Morita T, *et al.* Interleukin-1 increases the production of endothelin-1 by cultured endothelial cells. *Biochem Biophys Res Commun* 1990; **166:** 324–329.
6. Yoshizumi M, Kurihara H, Sugiyama T, *et al.* Hemodynamic Shear Stress Stimulates Endothelin Production by Cultured Endothelial Cells. *Biochem Biophys Res Commun* 1989; **161:** 859–864.
7. Harlan JM, Harker LA, Reidy MA, Gajdusek CM, Schwartz SM, Striker GE. Lipopolysaccharide-mediated bovine endothelial cell injury *in vitro*. *Lab Invest* 1983; **48:** 269–274.
8. Yamada O, Moldow CF, Sacks T, Craddock PR, Boogaerts MA, Jacob HS. Deleterious effects of endotoxin on cultured endothelial cells: an *in vitro* model of vascular injury. *Inflammation* 1981; **5:** 115–126.
9. Sugiura M, Inagami T, Kon V. Endotoxin stimulates endothelin release *in vivo* and *in vitro* as determined by radioimmunoassay. *Biochem Biophys Res Commun* 1989; **161:** 1220–1227.
10. Beutler BA, Milsark IW, Cerami A. Cachetin/tumour necrosis factor: production, distribution, and metabolic fate *in vivo*. *J Immunol* 1985; **135:** 3972–3977.
11. Beutler BA, Milsark IW, Cerami A. Passive immunization against cachetin/tumour necrosis factor protects mice from lethal effect of endotoxin. *Science* 1985; **229:** 868–871.
12. Bertani T, Abbate M, Zoja C, Corna D, Remuzzi C. Sequence of glomerular changes in an experimental endotoxemia: a possible model of hemolytic uremic syndrome. *Nephron* 1989; **53:** 330–337.
13. Bertani, T, Abbate M, Zoja C, *et al.* Tumour necrosis factor induces glomerular damage in the rabbit. *Am J Pathol* 1989; **134:** 419–430.
14. Zoja C, Furci L, Ghilardi F, Zilio P, Benigni A, Remuzzi G. Cyclosporin-induced endothelial cell injury. *Lab Invest* 1986; **55:** 455–462.
15. Jaffe EA, Hoyer LW, Nachman RL. Synthesis of antihemophilic factor antigen by cultured human endothelial cells. *J Clin Invest* 1973; **52:** 2757–2764.
16. Rambaldi, A, Young DC, Griffin JD. Expression of the M-CSF (CSF-1) gene by human monocytes. *Blood* 1987; **69:** 1409–1413.
17. Zoja C, Orisio S, Perico N *et al.* Constitutive expression of endothelin gene in cultured human mesangial cells and its modulation by transforming growth factor-$\beta$, thrombin, and a thromboxane $A_2$ analogue. *Lab Invest* 1991; **64:** 16–20.
18. Feinberg AP, Volgestein B. A technique for radiolabeling DNA restriction endonuclease fragments to high specific activity. *Anal Biochem* 1983; **132:** 6–13.
19. Minty A, Caravatti M, Robert B *et al.* Mouse actin messengers RNAs. Construction and characterization of a recombinant plasmid molecule containing a complementary DNA transcript of mouse $\alpha$-actin mRNA. *J Biol Chem* 1981; **256:** 1008–1014.
20. Pober JS. Cytokine-mediated activation of vascular endothelium: physiology and pathology. *Am J Pathol 1988;* **133:** 426–433.
21. Brenner BM, Troy JL, Ballermann BJ. Endothelium-dependent vascular responses. *J Clin Invest* 1989; **84:** 1373–1378.
22. Lamas S, Michel T, Brenner BM, Marsden PA. EDRF synthesis by endothelial cells: evidence for a pathway induced by TNF-$\alpha$. *Am J Physiol* 1991; **261:** C634–C641.
23. Tracey KJ, Beutler B, Lowry SF *et al.* Shock and tissue injury induced by recombinant human cachetin. *Science* 1986; **234:** 470–474.
24. Mathison JC, Wolfson E, Ulevitch RJ. Participation of tumor necrosis factor in the mediation of gram negative bacterial lipopolysaccharide-induced injury in the conscious rabbit. *J Clin Invest* 1988; **81:** 1925–1937.
25. Weinberg JR, Wright DJM, Guz A. Interleukin-1 and tumor necrosis factor cause hypotension in the conscious rabbit. *Clinical Sci* 1988; **75:** 251–255.
26. Miller WL, Redfield MM, Burnett JC. Integrated cardiac, renal, and endocrine actions of endothelin. *J Clin Invest* 1989; **83:** 317–320.
27. King AJ, Brenner BM, Anderson S. Endothelin: a potent renal and systemic vasoconstrictor peptide. *Am J Physiol* 1989; **256:** F1051–F1058.
28. Badr KF, Murray JJ, Breyer MD, Takahashi K, Inagami T, Harris RC. Mesangial cells, glomerular and vascular responses to endothelin in the rat kidney. *J Clin Invest* 1989; **83:** 336–342.
29. Perico N, Dadan J, Gabanelli M, Remuzzi G. Cyclooxygenase products and atrial natriuretic peptide modulate the renal response to endothelin. *J Pharmacol Exp Ther* 1990; **252:** 1213–1220.
30. Thiemermann C, Lidbury P, Thomas R, Vane J. Endothelin inhibits *ex vivo* platelet aggregation in the rabbit. *Eur J Pharmacol* 1988; **158:** 181–182.

ACKNOWLEDGEMENTS. We thank Dr Luca Benatti, Farmitalia Carlo Erba Milan, Italy, for kindly providing us with the human preproendothelin-1 probe.

**Received 4 May 1992; accepted in revised form 11 June 1992**
